# A systematic review and meta-analysis of dietary fat effects on reproductive performance of sows and growth performance of piglets

**DOI:** 10.1186/s40104-021-00662-3

**Published:** 2022-02-08

**Authors:** Lixue Wang, Shuai Zhang, Lee J. Johnston, Crystal L. Levesque, Jingdong Yin, Bing Dong

**Affiliations:** 1 State Key Laboratory of Animal Nutrition, College of Animal Science and Technology, China Agricultural University, Beijing, 100193 China; 2 West Central Research and Outreach Center, University of Minnesota, Morris, MN 56267 USA; 3 Department of Animal Science, College of Agriculture and Biological Sciences, South Dakota State University, Brookings, SD 57007 USA

**Keywords:** Diets, Fat, Growth performance, Piglets, Reproductive performance, Sows

## Abstract

Effects of added fat in sow diets on the sows’ reproductive performance and offspring growth performance are influenced by multiple factors such as genetics, nutrition, parity, ambient temperatures, and farm management. Individual studies cannot cover all these factors. With the view to address this challenge, we searched all studies that were published from 1986 to 2020, and performed a systematic review and meta-analysis on the fat effect. In total, 19 papers were collected and analyzed. Fat supplementation in sow diets during late gestation and lactation decreased average daily feed intake (ADFI, *P* < 0.05) and tended to increase average daily energy intake (ADEI, *P* = 0.11). It had no impact on litter weights at birth (*P* = 0.40) or weaning (*P* = 0.46). It increased total numbers of piglets at birth (*P* = 0.07), but had no effect on liveborn per litter (*P* = 0.90) or survival rate (*P* = 0.48) of piglets to weaning. Fat supplementation had no significant effect on sow body weight loss (*P* = 0.67) or backfat thickness changes (*P* = 0.66), but sows fed diets with added fat had increased milk fat concentration (*P* = 0.03) and shorter wean to estrus intervals (WEI, *P* = 0.01). In specific circumstances, fat supplementation tended to improve growth performance of piglets with low litter weights at birth (*P* = 0.14), or when the sows lost large amounts of body weight during lactation (*P* = 0.11). The level of supplemented fat was 10% and higher would decrease liveborn per litter at neutral temperature (*P* = 0.10). The meta-analysis revealed that fat supplementation to sows diet during late gestation and lactation can be beneficial for sow reproductive performance and litter growth performance.

**Supplementary Information:**

The online version contains supplementary material available at 10.1186/s40104-021-00662-3.

## Introduction

Genetic selection and improvements in health, management and nutrition have led to dramatic increases in sow productivity [1, 2]. With improved productivity, sows often mobilize body reserves to meet energy requirements during gestation and lactation [3]. Adding fat to sow diets is one potential approach to ensure sows and piglets consume sufficient energy. Over the last two to three decades, many studies have evaluated effects of fat supplementation in diets on reproductive performance of sows and growth performance of piglets [4–32]. We conducted a systematic review to evaluate effects of fat supplementation in sow diets on sow reproductive performance and litter growth performance using these studies.

Effects of added fat on sows’ reproductive performance and growth performance of offspring are inconsistent among studies because several factors such as genetics, nutritional supplementation, study parity of the sow, environmental temperatures, health status of sows and farm management likely influence results. Individual studies cannot standardize all these factors. To address this challenge, studies published from 1986 to 2020 that considered effects of dietary fat on sow feed intake, sow performance, and litter performance were retrieved. With these studies, we conducted a systematic review and meta-analysis to determine effects of dietary fat for sows. The aim of this review was to reveal the effects of added fat on sows and piglets across fat types, genetics, sow parities, dietary supplementation levels, and management systems. In total, 19 papers were included in the meta-analysis. This systematic review and analysis provide meaningful information to aid nutritionists and pig farmers effectively utilize dietary fat supplementation to improve sow performance and piglet growth.

## Materials and methods

### Databases queried

We searched ISI Web of Science, Science Direct, Scopus, ProQuest and Pubmed databases for publications related to use of dietary fat in sow diets. The following keywords or their combinations: high fat, high lipids, oil, dietary fat, sow and sows uncovered 29 papers [4–32] published from 1986 to 2020.

### Criteria for study selection

For a study to be included in the meta-analysis, all of the following criteria were satisfied. We screened these studies according to the following criteria:

1) The article was published in a peer-reviewed journal.

2) The study contained an un-supplemented treatment as a control and a fat supplemented group as a treatment(s).

3) The period of dietary fat supplementation occurred during late gestation (later than gestation day 85) to lactation or during lactation.

4) Supplementation levels of fat were at least equal to 2% (w/w) in order to exhibit fat effect. The reason for the supplementation level is that according to National Research Council (NRC), background fat level without additional fat supplementation is approximately 2%.

5) The author reported on at least one of the following response criteria: Average daily feed intake (ADFI) of sows during lactation, average daily energy intake (ADEI) of sows during lactation, litter weight at birth (Litter WT/birth), litter weight at weaning (Litter WT/wean), litter average daily gain during lactation (Litter ADG), liveborn per litter (No. born alive), litter size at weaning (No. weaned), survival rate of piglets from birth to weaning (Survival rate), change in sow body weight during lactation (sow Δ-WT), change in sow backfat thickness during lactation (sow Δ-BF), wean to estrus interval of sows (sow WEI), and milk fat concentration (Milk fat).

In these 29 papers, dietary fat supplementation was less than 2% in five papers [22, 29–32], the fat supplementation period was not in late gestation or lactation in four papers [16, 21, 23, 25], and one article was not peer reviewed [26], which led to exclusion of five articles from the final review. Therefore, 19 papers were included in this systematic review and meta-analysis (Table 1).

**Table 1:** Summary of articles used for meta-analysis

Authors	Suppl. period^1^	Treat. No.^2^	Fat level^3^, %	Fat type	ADFI, kg/d	ADEI, Mcal/d	Litter size (birth)	Liveborn litter size	Litter WT^4^ (birth), kg	Litter WT^4^ (wean), kg	Litter ADG, kg/d	Litter size (wean)	Survival rate^5^,%	Sow Δ-WT^6^ (farr-wean), kg	Sow Δ-BF^7^ (farr-wean), mm	Sow WEI^8^, d	Milk fat concentration, %	Temp^9^
[4] Tilton et al. 1999	G110~L28		C: 0		6.21±1.23	19.99±4.3			15.54±1.86	58.76±7.1	2.06±0.25		96.0±10.6	-2.18±11.45	-1.01±1.9		7.72±0.86	Neutral
		1	F: 10%	Tallow	5.73±1.23	21.00±4.3			15.02±1.86	57.34±7.1	2.02±0.25		92.1±10.6	-1.73±11.45	0.45±1.9		9.59±0.86	
	G110~L21		C: 0		5.04±0.58	16.43±2.4			14.96±1.35	58.19±3.8	2.06±0.12							
		2	F: 10%	Tallow	4.97±0.58	18.49±2.4			14.48±1.35	61.96±3.8	2.26±0.12							Neutral
[5] Jin et al. 2017	G90~L21		C: 0		5.15±0.21	16.37±0.23	10.85±2.24	10.35±2.13			1.629±0.39		91.23±6.75		-3.71±0.61	5.56±1.65	5.88±0.78	Neutral
		1	F1: 4.13%	Palm oil	4.91±0.21	16.41±0.23	10.86±2.24	10.33±2.13			1.796±0.39		93.09±6.75		-3.14±0.61	4.95±1.65	6.35±0.78	
		2	F2: 3.89%	Fish oil	5.00±0.21	16.69±0.23	11.64±2.24	11.36±2.13			2.160±.39		96.81±6.75		-3.48±0.61	4.31±1.65	6.56±0.78	
		3	F3: 3.78%	Soybean oil	4.95±0.21	16.59±0.23	11.64±2.24	11.45±2.13			1.965±0.39		96.82±6.75		-3.36±0.61	4.37±1.65	7.15±0.78	
[6] Shurson and Irvin 1992	L1~L28		C: 0		3.393±1.288	10.72±4.072	9.85±2.4	9.00±2.36	14.1±3.2	59.1±2.8	1.607±0.68		71.1±30.7	-10.7±18.4	-5.0±5.6		6.12±1.74	Neutral
		1	F: 10%	Corn oil	3.096±1.247	11.02±4.438	8.65±2.32	7.99±2.36	13.0±3.2	56.4±2.8	1.550±0.68		76.7±30.7	-12.3±20.9	-4.0±5.4		7.84±1.74	
			C: 0		3.925±0.73	12.403±2.32	10.33±3.0	9.66±2.9	14.6±4.2	63.7±19.7	1.754±0.56		83.6±25.4	-19.0±21.4	-4.0±4.2	11.2±8.2	6.51±2.68	Neutral
		2	F: 10%	Corn oil	3.843±0.71	13.681±2.54	9.7±3.26	9.29±2.9	14.6±4.2	63.7±19.7	1.754±0.56		80.9±25.4	-9.5±22.1	-3.1±4.8	9.2±8.2	8.18±2.68	
[7] Azain MJ 1993	G91~L7		C: 0		2.957±0.66	10.0±2.25	16.81±1.79	11.33±2.33	13.07±3.26	46.96±9.9	1.61±0.31		80.2±11.87				8.78±2.2	Neutral
		1	F1: 12%	Soybean oil	3.243±0.66	12.0±2.57	16.41±1.79	10.79±2.33	13.03±3.26	48.53±9.9	1.69±0.31		81.2±11.87				9.62±2.2	
		2	F2: 12%	10%MCT+2%soybean oil	3.286±0.66	12.37±2.43	17.1±1.79	11.59±2.33	14.31±3.26	58.87±9.9	2.12±0.31		90.3±11.87				7.62±2.2	
[8] Averette et al. 1999	G90~L21		C: 0		4.42±1.48	14.608±4.8	16.14±2.64	14.43±3.46			2.26±0.07		85.97±16.4	-0.98±4.06			7.8±1.9	Neutral
		1	F: 10%	Choice white grease	4.19±1.48	15.674±5.5	15.83±2.64	11.67±3.46			2.24±0.07		78.70±16.4	-7.15±4.06			10±1.9	
			C: 0		4.85±1.48	16.029±4.8	12±2.64	11.5±3.46			2.25±0.07		83.89±16.4	-7.72±4.06				
		2	F: 10%	Choice white grease	4.49±1.48	16.797±5.5	11.33±2.64	10.33±3.46			2.69±0.07		88.66±16.4	-4.16±4.06				
			C: 0		4.36±1.48	14.41±4.8	9.84±2.64	9.5±3.46			1.816±0.07		96.48±16.4	2.43±4.06				
		3	F: 10%	Choice white grease	4.00±1.48	14.96±5.5	10.34±2.64	9.67±3.46			1.736±0.07		91.03±16.4	-3.93±4.06				
			C: 0		4.78±1.48	15.80±4.8	10.34±2.64	10.00±3.46			2.79±0.07		92.82±16.4	-0.88±4.06				
		4	F: 10%	Choice white grease	4.96±1.48	18.56±5.5	10.33±2.64	9.83±3.46			2.428±0.07		88.43±16.4	-7.43±4.06				
[9] Christon et al. 1999	G105~L28		C: 0		6.2±0.84	18.46±2.76	14.4±1.41	13.6±1.41	15.8±1.41	92.4±6.79	2.8±0.28		77.9±11.3	-19.6±8.2	1.6±0.14			Neutral
		1	F1: 6%	Peanut-rapeseed oil:50:50	5.6±0.84	18.73±2.76	13.8±1.41	13.2±1.41	14.9±1.41	86.7±6.79	2.7±0.28		80.3±11.3	-14.0±8.2	4.5±0.14			
		2	F2: 12%		5.2±0.84	19.50±2.76	12.5±1.41	12.1±1.41	16.1±1.41	82.1±6.79	2.4±0.28		82.6±11.3	-13.0±8.2	-0.7±0.14			
			C: 0		4.8±0.84	14.14±2.76	11.6±1.41	9.6±1.41	11.3±1.41	60.4±6.79	1.8±0.28		79.2±11.3	-8.6±8.2	-5.6±0.14			High
		3	F1: 6%	Peanut-rapeseed oil:50:50	5.7±0.84	18.60±2.76	10.2±1.41	10.1±1.41	12.7±1.41	63.4±6.76	1.9±0.28		82.0±11.3	-16.8±8.2	-5.6±0.14			
		4	F2: 12%		4.5±0.84	16.48±2.76	12.1±1.41	10.3±1.41	13.5±1.41	67.0±6.79	2.0±0.28		86.4±11.3	-15.4±8.2	-7.8±0.14			
[10] Gatlin et al. 2002	G90~L15.5		C: 0		7.19±1.28	23.62±4.2	11.3±4.48	10.78±3.07			1.79±2.94		94.8±11.5	0±13.83	4.56±1.01	6.3±5.12		Neutral
		1	F1: 10%	MCT	6.41±1.26	23.79±4.67	11.47±4.41	10.55±3.07			1.87±2.9		94.2±11.3	0.155±13.48	4.39±9.96	5.84±5.04		
		2	F2: 10%	choice white grease	6.54±1.26	24.28±4.67	11.44±4.41	10.73±3.02			1.88±2.9		93.9±11.3	0.155±13.48	3.57±9.96	6.59±5.04		
[11] Schoenherr et al. 1989	G100~L22		C: 0		5.90±0.82	19.4±3.12					1.88±0.31		91.0±9	2.6±1.3		7.0±3		Neutral
		1	F1: 10.65%	choice white grease	5.31±0.82	20.0±3.12					1.98±0.33		93.8±9	2.0±1.3		4.1±3		
			C: 0		3.36±1.84	10.9±1.84					1.64±0.27		94.7±9	15.9±9.1		5.4±2.9		High
		2	F2: 10.65%	choice white grease	3.54±1.84	13.0±1.84					1.79±0.30		94.9±9	16.3±9.1		4.8±2.9		
[12] Christon et al. 2005	G105~L28		C: 0%		6.2±1.8	18.59±3.51	14.4±3.2	13.6±2.8	15.8±2.1	92.4±17.2	2.8±0.6	10.6±0.7	77.9±2.5	-19.7±3.8	1.6±0.4		7.5±0.4	Neutral
		1	F1: 6%	Peanut oil+sugarbeet oil:50:50	5.6±1.2	18.73±2.58	13.8±2.7	18.3±2.5	14.9±3.0	86.7±14.6	2.7±0.4	10.6±0.7	57.9±2.5	-13.7±7.8	4.5±0.9		5.87±0.4	
		2	F2: 12%		5.2±1.6	19.52±5.13	12.5±3.3	12.1±2.8	16.1±3.3	82.1±14.5	2.4±0.4	10.1±0.8	83.5±2.5	-13.0±9.0	-10.7±2.1		12.1±0.4	
			C: 0%		4.8±1.3	14.14±3.7	11.6±5.5	9.6±3.9	11.3±3.8	60.6±19.0	1.8±0.6	7.6±1.7	79.2±0.4	-7.6±2.0	-5.6±1.2		6.3±0.2	High
		3	F1: 6%	Peanut oil+sugarbeet oil:50:50	5.7±0.9	18.63±2.89	10.2±2.6	10.0±2.4	12.7±3.3	63.4±15.2	1.9±0.5	8.2±1.7	82±0.4	-15.9±2.1	-5.6±0.9		7.5±0.2	
		4	F2: 12%		4.5±0.8	16.48±2.82	12.1±3.2	10.3±3.3	13.5±3.0	67±12.7	2.0±0.4	8.9±1.2	86.4±0.4	-15.4±2.0	-7.8±1.2		8.44±0.3	
[13] Wang et al. 2017	G107~L21		C: 0		5.75±0.66	17.01±1.8	11.47±0.96	10.87±2.88	16.57±1.68	60.05±5.8	2.07±0.2		93.0±17.8				6.20±1.2	Neutral
		1	F: 3%	Soybean oil	5.47±0.66	17.03±1.8	11.46±0.96	10.92±2.88	16.28±1.68	57.66±5.8	1.97±0.2		94.0±17.8				6.29±1.2	
[14] Ma et al. 2020	G107~L24		C: 0		6.91±1.23	22.8±2.84	10.2±2.12	10.02±2.1	16.18±4.0	70.71±6.0	2.21±0.33		93.45±6.48	-33.43±20.1	-1.55±3.1		5.21±0.8	Neutral
		1	F: 8%	Soybean oil	5.39±1.23	20.1±3.08	10.58±2.12	10.58±2.1	18.01±4.0	74.72±6.0	2.41±0.33		98.93±6.48	25.64±20.1	-1.51±3.1		5.95±0.8	
[15] Neal et al. 1999	G105~L28		C: 0		3.9±0.36	12.68±1.17	11.52±4.61	9.76±4.4	13.078±0.36	60.449±1.71	1.692±0.05		89.2±7.3	-10.64±5.0	-3.02±0.16			Neutral
		1	F1: 3%	Low acid yellow fat	3.63±0.37	12.30±1.25	11.56±4.67	9.78±4.4	13.594±0.36	59.84±1.71	1.65±0.05		82.38±7.3	-11.5±5.0	-1.83±0.09			
		2	F2: 6%		3.86±0.36	13.61±1.26	11.56±4.49	9.78±4.4	13.105±0.36	58.879±1.56	1.635±0.05		83.5±7.3	-7.0±3.0	-1.83±0.08			
		3	F3: 9%		3.67±0.35	13.41±1.27	11.36±4.37	9.52±4.4	12.566±0.36	57.642±1.56	1.61±0.05		80.3±7.3	-11.76±5.0	-1.98±0.08			
[17] Lauridsen et al. 2004	G108~L28		C: 0		6.04±4.55	19.41±1.65	12.2±0.29	11.3±2.5			2.068±0.48		89.2±11.5	6.7±12.5			6.5±1.04	Neutral
		1	F1: 8%	Animal fat	5.85±4.55	21.09±1.65	13±0.29	11.6±2.5			2.453±0.48		95.0±11.5	6.4±12.5			7.1±1.04	
		2	F2: 8%	Rapeseed oil	5.82±4.55	20.95±1.65	13.2±0.29	12.1±2.5			2.279±0.48		88.0±11.5	4.1±12.5			6.4±1.04	
		3	F3: 8%	Fish oil	5.81±4.55	21.14±1.65	12.4±0.29	10.8±2.5			2.118±0.48		88.1±11.5	2.7±12.5			6.5±1.04	
		4	F4: 8%	Coconut oil	5.83±4.55	20.93±1.65	14±0.29	12.7±2.5			2.368±0.48		90.0±11.5	4.6±12.5			7.5±1.04	
		5	F5: 8%	Palm oil	5.9±4.55	21.1±1.65	12.7±0.29	11.7±2.5			2.371±0.48		89.4±11.5	3.5±12.5			7.1±1.04	
		6	F6:8%	Sunflower oil	5.83±4.55	21.36±1.65	14.1±0.29	12.5±2.5			2.454±0.48		90.2±11.5	11.0±12.5			6.9±1.04	
[18] Rosero et al. 2012	G110±2~L19±2		C: 0		4.08±1.19	13.22±3.85		11.67±3.13			2.11±2.38		89.79±23.8	-1.61±24.7	-0.36±0.36	9.2±7.33		High
		1	F1: 2%	Animal-vegetable fat	4.18±1.2	13.96±3.96		11.84±3.13			2.20±2.4		89.04±23.8	-2.27±24.7	-0.31±0.36	7.8±7.33		
		2	F2: 4%		4.44±1.19	15.23±4.12		11.55±3.13			2.13±2.38		89.22±23.8	-0.76±24.7	-0.29±0.36	7.9±7.33		
		3	F3: 6%		4.34±1.19	15.32±4.13		11.69±3.13			2.16±2.38		88.58±23.8	-2.20±24.7	-0.26±0.36	8.0±7.33		
[19] Rosero et al. 2012	G110±2~L22±2		C: 0		4.2±0.96	13.71±3.41	12.98±3.12	11.76±2.81			2.074±0.37		89.0±1.11	-0.27±0.81				High
		1	F1: 2%	Animal-vegetable fat	4.36±0.96	14.64±3.41	13.14±3.35	11.90±3.01			2.098±0.37		90.2±1.11	-0.17±0.81				
		2	F2: 4%		4.5±0.96	15.52±3.41	13.96±3.16	12.96±2.84			2.039±0.37		88.6±1.11	-0.24±0.81				
		3	F3: 6%		4.46±0.96	15.81±3.41	14.06±3.32	13.05±2.98			2.137±0.37		90.7±1.11	-0.17±0.81				
			C: 0		4.2±0.96	13.71±3.41	12.98±3.12	11.76±2.81			2.074±0.37		89.0±1.11	-0.27±0.81				High
		4	F4: 2%	Choice white grease	4.36±0.96	14.62±3.41	12.32±3.32	11.20±2.98			2.209±0.37		91.5±1.11	-0.05±0.81				
		5	F5: 4%		4.38±0.96	15.07±3.41	13.72±3.2	12.41±2.88			2.103±0.37		87.1±1.11	-0.11±0.81				
		6	F6: 6%		4.26±0.96	15.06±3.41	14.03±3.39	12.99±3.05			2.101±0.37		87.5±1.11	0.11±0.81				
[20] Shurson et al. 1986	G107~L28		C: 0		4.54±1.06	14.18±4.27	10.2±2.8	9.4±2.53	14.7±4.22	48.9±14.5	1.62±0.5		88.4±2.0	-10.8±4.9		9.69±10.72	8.8±1.91	Neutral
		1	F: 10%	Dried fat	4.57±1.06	15.84±4.27	10.9±2.8	10.2±2.53	16.8±4.22	56.1±14.5	1.87±0.5		88.0±2.0	-11.2±4.9		7.25±10.72	11.2±1.91	
[24] Coffey et al. 1987	L0~L21		C: 0		4.5±0	14.3±0	10.86±2.71	9.64±2.7			1.47±0.36		89.1±13.2	-14.49±8.0			4.89±2.17	Neutral
		1	F: 10%	Vegetable oil	5.0±0	18.385±0	10.09±2.71	9.9±2.7			1.48±0.36		87.0±13.2	-12.84±8.0			5.23±2.17	
[27] Rosero et al. 2015	G110~L17		C: 0														6.3±0.85	
		1	F1: 6%	Animal-vegetable fat													6.7±0.85	Neutral
		2	F2: 6%	Choice white grease													7.1±0.85	
[28] Leonard et al. 2010	G109~L26		C: 0														8.91±1.23	Neutral
		1	F1: 2%	Fish oil													8.43±1.23	

Note: ^1^Period of fat supplementation. *G*: Gestation period; *L*: Lactation Period. ^2^Treat. No.: The No. of a treatment in a study. In forest plots in following analysis, one treatment was labelled as Reference No. + Treatment No. ^3^Fat level: Levels of supplemental fat; “C” represents un-supplemented controls; “F” represents fat supplemented treatments; Numbers represent treatment No. ^4^Litter weight at birth or at weaning. ^5^Survival rate was calculated by litter size at weaning divided by litter size after cross-foster within 24-48 h after farrowing as indicated in articles (except for [10] which performed cross-fostering after 72 hrs postpartum). ^6^Sow Δ-WT: Change of sow body weight from farrowing to wean. Negative value meant loss while positive value meant gain. ^7^Sow Δ-WT: Change of sow backfat thickness from farrowing to wean. Negative value meant loss while positive value meant gain. ^8^Sow weaning to estrus interval (sow *WEI*). ^9^Temp.: Environmental temperature during studies; “High” represents the study was conducted at high temperature; “Neutral” represents at neutral temperature. Other abbreviations: Average daily feed intake during lactation period (*ADFI*); Average daily energy intake during lactation period (ADEI); Litter average daily gain during lactation (Litter *ADG*), Total piglet numbers per litter at birth (Litter size (birth)); Total live piglet numbers per litter at birth (Liveborn litter size); Litter size at weaning (Litter size (wean)). MCT: medium chain triglyceride. All values, expressed as mean±standard deviation (*MEAN±SD*), are either reported in the text of articles or calculated by available parameters in articles

### Definition

#### Study, treatment, and observation

In this review, the term, “study”, refers to a scientific article which involves one or more treatments. The term, “treatment”, refers to a comparison of a treated group of sows fed supplemental fat and its corresponding un-supplemented control group of sows. One study can have several treatments, as long as a comparison between an un-supplemented control and a fat supplemented treatment exists. An observation refers to a measurable dependent response variable.

#### ADFI and ADEI

During late gestation, sows were fed a fixed amount of feed based on body weight or a target total energy intake. During lactation, sows were provided ad libitum access to feed. Thus, we analyzed ADFI and ADEI only during the lactation period (post farrowing to weaning). Most authors reported metabolizable energy (ME) concentration of diets except for two studies [5, 14] in which authors reported digestible energy (DE). As long as the control was an un-supplemented group, studies using both ME and DE were deemed valid.

#### Litter ADG

Litter ADG was either reported in the article, or calculated by subtracting litter weight at weaning from litter weight at birth (after cross-fostering) then divided by days of lactation.

#### Piglet survival rate

Piglet survival rate was either reported in the article, or calculated as number of piglets per litter at weaning divided by number of piglets per litter (after cross-fostering) multiplied by 100%.

#### Changes of sow body weight and backfat thickness

Change of sow body weight and backfat thickness was either reported in the article, or calculated by subtracting the sow body weight or backfat thickness at weaning from its body weight or backfat thickness at farrowing. Negative values indicate loss of body weight or backfat thickness during lactation while positive values indicate gain of body weight or backfat thickness.

#### Milk fat concentration

Milk fat concentration was defined as the percentage of fat in milk (%, w/v). The milk samples were collected from d 7 to d 21 of lactation in these studies. Milk fat was determined by extraction with organic solvents [4, 9, 20, 24, 26, 27] or by an automated infrared filtration system [17, 25].

#### Litter size

Total born litter size includes number of live, stillborn, and mummified piglets. Liveborn per litter contains only number of live pigs at birth.

#### Temperature

When authors claimed that the studies were conducted at tropical environment or high temperature, these studies (treatments) were categorized as “High temperature”. There were 5 studies [9,11,12,18,19] categorized “high temperature”. The studies of [9] and [12] described “high temperature (tropical environment)” by providing geographic location and season. The study of [11] provided a specific high temperature (32 °C) versus a neutral temperature (20 °C). The studies of [18] and [19] were conducted in Oklahoma and reported tropical environmental temperatures at 33 ± 5 °C. The rest of the studies either did not clearly describe the temperatures during studies, or reported average temperature under 30 °C.

### Statistical analysis

In this study, R software version 4.1.0 (“Camp Pontanezen” Copyright © 2021; The R Foundation for Statistical Computing) was used to analyze all studies using the meta package. Table 2 shows the code in R for this meta-analysis. The effects of dietary fat on sow performance and litter growth performance were determined by comparing the dietary fat treatment with the corresponding un-supplemented control. Before conducting meta-analysis, we tested heterogeneity among studies by calculating *Cochran-Q*, *I*^*2*^ statistics, and *chi-square* test with significance set as *P* < 0.05 and *I*^*2*^ statistics with a cut-off of 50% that defined statistical significance of heterogeneity. Due to the apparent heterogeneity among included studies, we chose a random-effects model for the meta-analysis. Forest plots were used for overall assessment of the meta-analysis. Probability values (*P* values) less than or equal to 0.10 were deemed significant and *P* values over 0.10 but less than 0.15 were considered a trend.

**Table 2:** R-code for meta-analysis of control and fat supplemented treatments

madata<-read.csv("F:/Meta/filename.csv",header = T)	
library (meta)	
m.res<-metacont (Ne,Me,Se,Nc,Mc,Sc,data=madata,studlab=paste (Reference), comb.fixed=F,comb.random=T, method.tau="SJ", hakn=T, sm="SMD")	
m.res	
forest(m.res, test.overall.random =T)	

To further investigate the potential factors such as temperature and supplementation level on the fat effect, we analyzed the study data through subgroup analysis. For example, when we analyzed ADFI, ADEI, litter ADG, Numbers of alive piglets at birth, Survival rate and sow body weight loss during lactation, we divided the studies into two subgroups according to the reported environmental temperature (high temperature or neutral temperature) because the observations in these variables were sufficient for statistical analysis compared to the rest variables. For analysis of other variables such as Milk fat concentration and WEI, we attempted to divide the data according to other factors, such as supplementation levels of fat, genetic lines, oil type, parities, but the numbers of the observations were not sufficient for a statistical analysis. Forest plots were used to demonstrate the fat effect on individual response variables.

## Results

### ADFI of sows

**Fig. 1:**
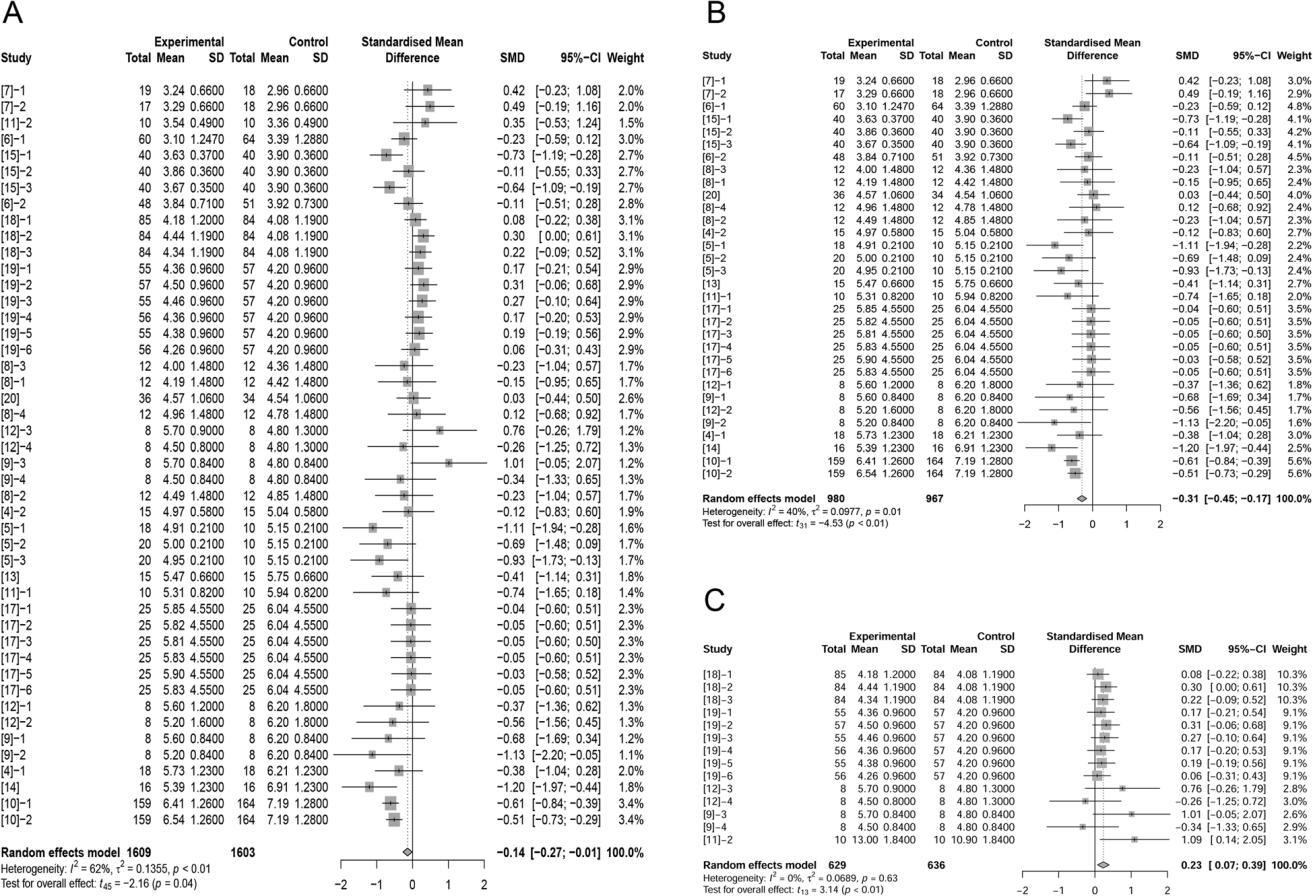
Forest plot showing effects of adding fat to diets during late gestation and lactation on ADFI (kg/d) of sows. Figure lists treatments in studies with treatment No. (Study), treatment group of fat-treatment or un-supplemented control (Experimental vs. Control), total observations (Total), mean of the treatment and standard deviation (Mean, SD), standardized mean difference (SMD), 95% confidence interval (95%-CI) and the weight accounting for the total statistics (weight). Test results for heterogeneity and overall fat supplementation effect are listed at lower left of each figure. *P* ≤ 0.10 was considered significant; 0.1 < *P* < 0.15 was considered a trend. **A**, Effect of adding fat on sow ADFI (all treatments); **B**, Effect of adding fat on sow ADFI at thermoneutral temperature; **C**, Effect of adding fat on sow ADFI at high temperature.

Authors of 16 papers (46 treatments) reported ADFI of sows was affected by adding fat in diets (1603 observations in un-supplemented control; 1609 observations in fat treatment). The standardized mean difference (SMD; “Difference” used in following text) between control and fat treatment was -0.14 kg/d (*P* = 0.04) (Fig. 1A), which means that inclusion of fat in diets decreased ADFI of sows. Further analysis (Supplemental Fig. 1) proved the relationship between fat levels and difference of ADFI.

Some authors reported increased ADFI due to fat supplementation at high temperature which was defined in Materials & Methods [9, 11, 12, 18, 19]. The treatments in these studies were recognized as high temperature treatments because authors claimed these studies were conducted in tropical environments. Consequently, we divided our studies into conducting at high temperature (14 treatments; 636 observations in control and 629 observations in fat treatment) and those conducting at neutral temperature (32 treatments; 967 observations in control and 980 observations in fat treatment). Fat inclusion decreased sows’ ADFI by 0.31 kg/d (*P* < 0.01) at neutral temperature (Fig. 1B). In contrast, high temperature conditions, dietary fat supplementation increased ADFI by 0.23 kg/d (*P* < 0.01; Fig. 1C).

### ADEI of sows

**Fig. 2:**
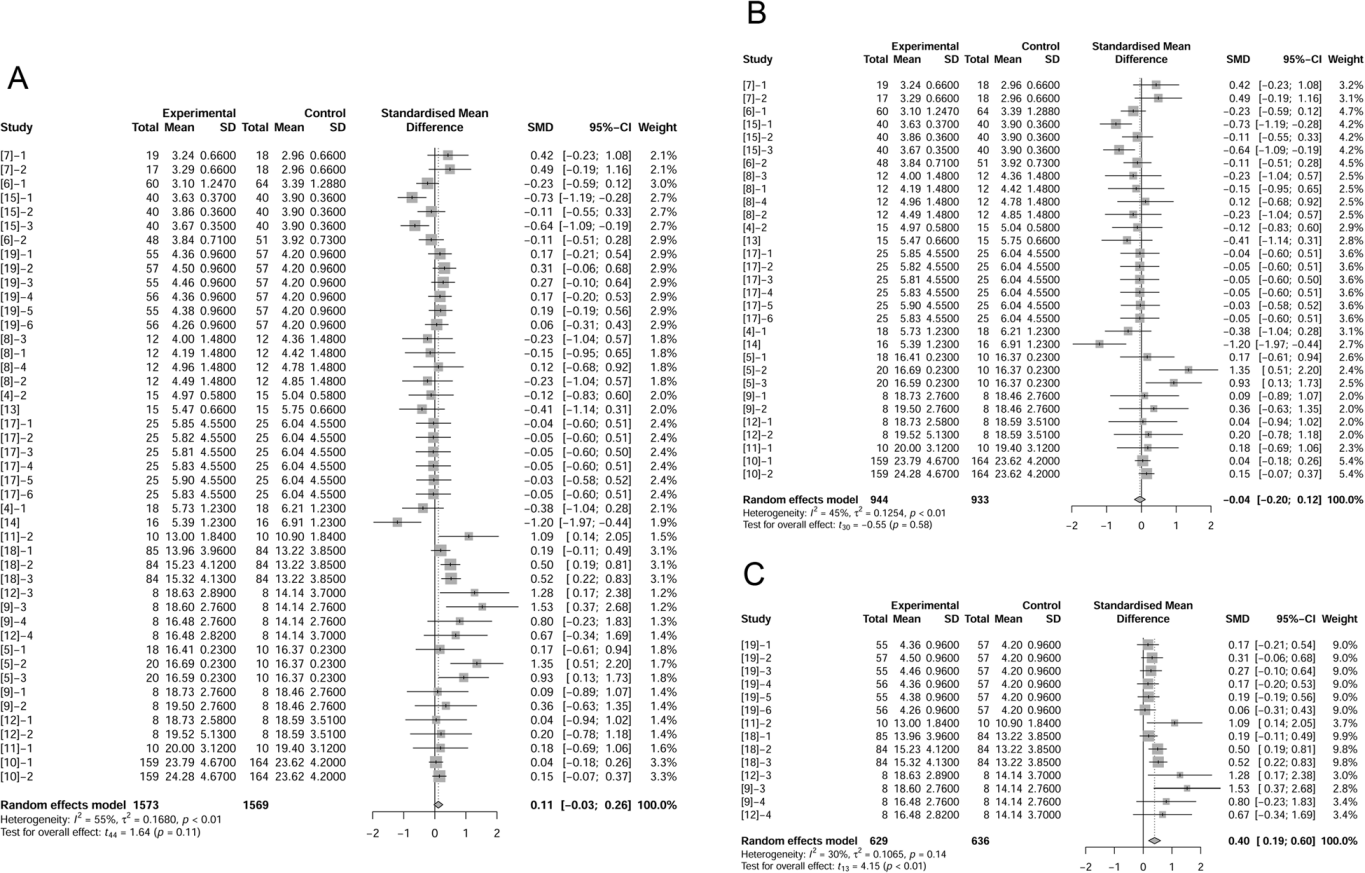
Forest plot showing effects of adding fat to diets during late gestation and lactation on ADEI (Mcal/d) of sows. Figure lists treatments in studies with treatment No. (Study), treatment group of fat-treatment or un-supplemented control (Experimental vs. Control), total observations (Total), mean of the treatment and standard deviation (Mean, SD), standardized mean difference (SMD), 95% confidence interval (95%-CI) and the weight accounting for the total statistics (weight). Test results for heterogeneity and overall fat supplementation effect are listed at lower left of each figure. *P* ≤ 0.10 was considered significant; 0.1 < *P* < 0.15 was considered a trend. **A**, Effect of adding fat on sow ADEI (all treatments); **B**, Effect of adding fat on sow ADEI at thermoneutral temperature; **C**, Effect of adding fat on sow ADEI at high temperature.

We further analyzed effects of fat inclusion on sows’ ADEI (Fig. 2 A, B, C). There were 19 papers (45 treatments) with 1569 observations in control and 1573 observations in fat treatment that reported fat effect on ADEI. The difference of ADEI between un-supplemented control and fat treatment was not statistically significant with a trend of increased ADEI of 0.11 Mcal/d (*P* = 0.11, Fig. 2A). When considering only studies conducted under neutral temperature conditions, ADEI was not different between control and fat treatments (*P* = 0.58, Fig. 2B). At high temperatures, fat supplementation increased ADEI by 0.4 Mcal/d with a 95% confidence interval (95%-CI) of 0.19 to 0.60 Mcal/d (*P* = 0.0012, Fig. 2C). Further analysis proved that fat addition with different levels decreased ADFI but tended to increase ADEI (Supplemental Fig. 1).

### Litter size and litter weight

**Fig. 3:**
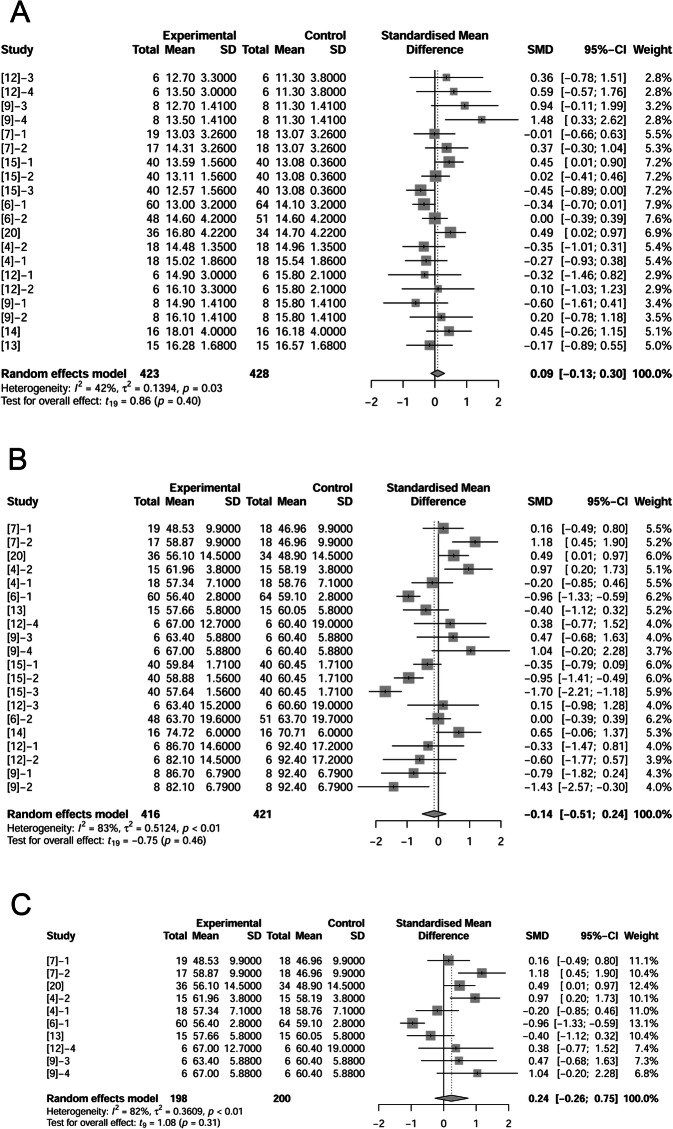
Forest plot showing effects of adding fat to diets during late gestation and lactation on litter weight (kg). Figure lists treatments in studies with treatment No. (Study), treatment group of fat-treatment or un-supplemented control (Experimental vs. Control), total observations (Total), mean of the treatment and standard deviation (Mean, SD), standardized mean difference (SMD), 95% confidence interval (95%-CI) and the weight accounting for the total statistics (weight). Test results for heterogeneity and overall fat supplementation effect are listed at lower left of each figure. *P* ≤ 0.10 was considered significant; 0.1 < *P* < 0.15 was considered a trend. A, Effect of adding fat on litter weight (at birth); B, Effect of adding fat on litter weight (at wean); C, Effect of adding fat on smaller litter weight than average (at birth).

Nine papers (19 treatments) reported litter weights (428 observations in control and 423 observations in fat treatment). There were no significant differences between control and fat treatment on litter weights at birth (*P* = 0.40; Fig. 3A) or at weaning (*P* = 0.46; Fig. 3B). Based on total litter weight at birth of the controls, we sorted data from lightest to heaviest. We analyzed the effects of fat supplementation on litter birth weight of the lightest 33% of litters. Fat supplementation tended to improve litter birth weight by 0.24 kg (*P* = 0.14) when litters were lighter than average at birth (Fig. 3C).

**Fig. 4:**
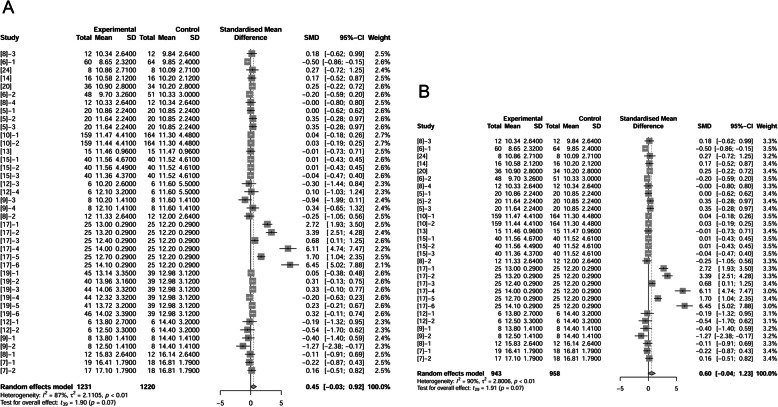
Forest plot showing effects of adding fat to diets during late gestation and lactation on litter size at birth. Figure lists treatments in studies with treatment No. (Study), treatment group of fat-treatment or un-supplemented control (Experimental vs. Control), total observations (Total), mean of the treatment and standard deviation (Mean, SD), standardized mean difference (SMD), 95% confidence interval (95%-CI) and the weight accounting for the total statistics (weight). Test results for heterogeneity and overall fat supplementation effect are listed at lower left of each figure. *P* ≤ 0.10 was considered significant; 0.1 < *P* < 0.15 was considered a trend. **A**, Effect of adding fat on litter size at birth (all treatments); **B**, Effect of adding fat on litter size at birth at thermoneutral temperature.

Litter weight was related closely to litter size, so we examined the effects of supplemental fat on litter size (Fig. 4A, B). Authors of 15 papers (39 treatments) reported number of total piglets (including mummies, stillborns and born alive) per litter at birth (1220 observations in control and 1231 observations in fat treatment). The difference between control and fat treatment was 0.45 piglets per litter (*P* = 0.07, Fig. 4A) which showed a positive effect of fat on total number of piglets at birth. Regression analysis on fat level and increased ME indicated a positive relationship (Supplemental Fig. 2). Environmental temperature may influence effects of fat on litter size, so we removed treatments at high temperature and re-analyzed the data. The difference between control and fat treatment at neutral temperature was 0.60 (*P* = 0.07, Fig. 4B). At high temperature, fat supplementation did not significantly change litter size at birth (data not shown).

**Fig. 5:**
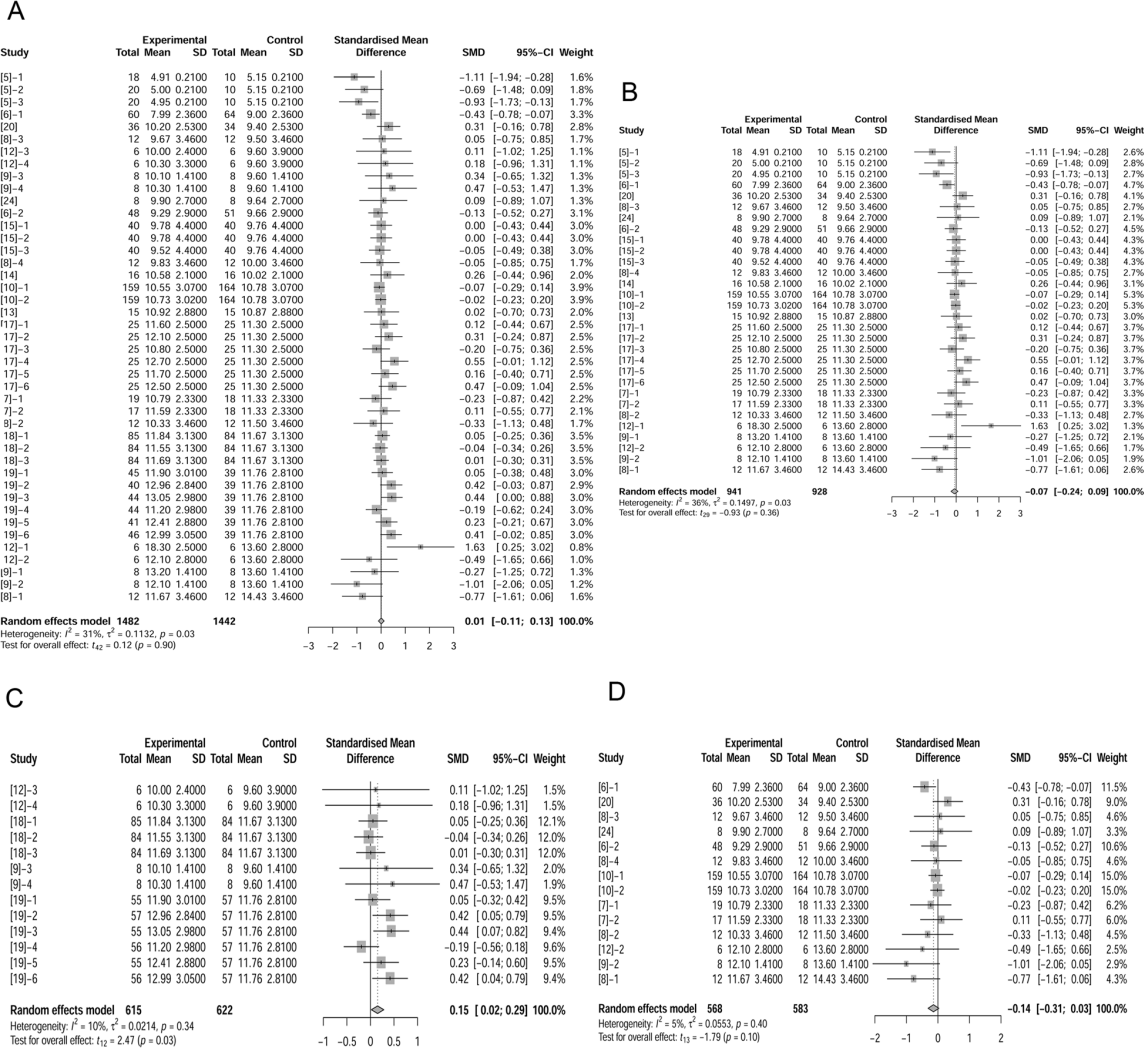
Forest plot showing effects of adding fat to diets during late gestation and lactation on number of alive piglets at birth. Figure lists treatments in studies with treatment No. (Study), treatment group of fat-treatment or un-supplemented control (Experimental vs. Control), total observations (Total), mean of the treatment and standard deviation (Mean, SD), standardized mean difference (SMD), 95% confidence interval (95%-CI) and the weight accounting for the total statistics (weight). Test results for heterogeneity and overall fat supplementation effect are listed at lower left of each figure. *P* ≤ 0.10 was considered significant; 0.1 < *P* < 0.15 was considered a trend. **A**, Effect of adding fat on number of alive piglets at birth (total); **B**, Effect of adding fat on number of alive piglets at birth at thermoneutral temperature; **C**, Effect of adding fat on number of alive piglets at birth at neutral temperature when fat level was equal to or over 10%.

However, when the number of liveborn per litter was analyzed in this review, fat supplementation showed no effect (*P* = 0.90) when comparing 1442 observations in control and 1482 observations in fat treatment (Fig. 5A). We confirm no significant effect of fat on liveborn per litter (*P* = 0.36, Fig. 5B) at neutral temperature, while at high temperature, fat supplementation had positive effect (increased 0.15 pigs per litter) on liveborn per litter (*P* = 0.03; Fig. 5C). The above analysis revealed that fat supplementation increased total number of piglets per litter at birth but had no significant effect on liveborn litter size.

To confirm the effect of fat on liveborn per litter, we further divided 29 treatments at neutral temperature into 2 categories: < 10% supplemental fat and ≥ 10% supplemental fat. When the fat level was ≥ 10%, fat decreased the liveborn per litter (*P* = 0.10; Fig. 5D). It demonstrated multiple functions of added fat on liveborn per litter: it increased the liveborn per litter at high temperature, but decreased it at neutral temperatures only if the supplementation level of fat was ≥ 10%.

### Litter ADG

**Fig. 6:**
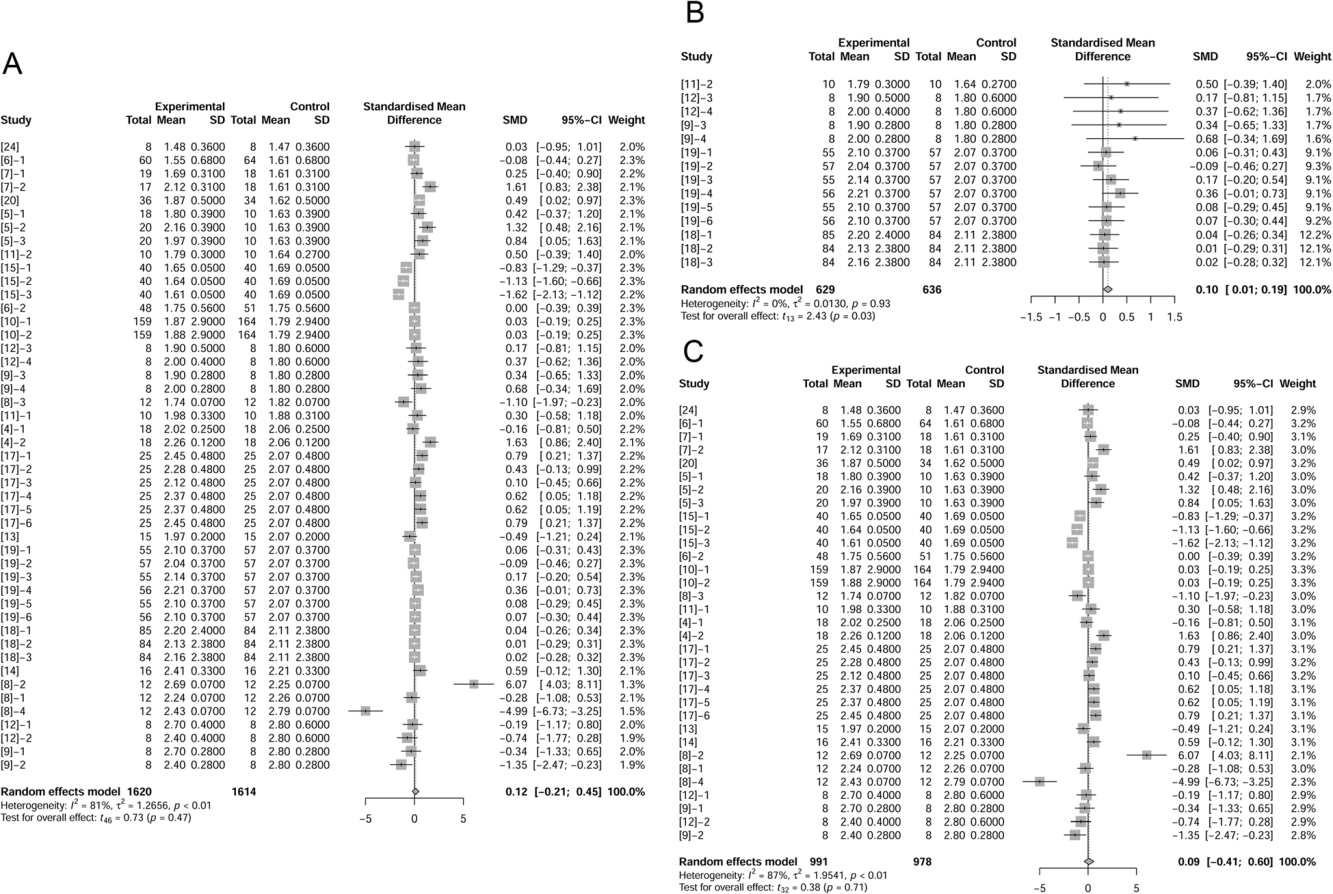
Forest plot showing effects of adding fat to diets during late gestation and lactation on ADG (kg) of piglets. Figure lists treatments in studies with treatment No. (Study), treatment group of fat-treatment or un-supplemented control (Experimental vs. Control), total observations (Total), mean of the treatment and standard deviation (Mean, SD), standardized mean difference (SMD), 95% confidence interval (95%-CI) and the weight accounting for the total statistics (weight). ADG, average daily gain. Test results for heterogeneity and overall fat supplementation effect are listed at lower left of each figure. *P* ≤ 0.10 was considered significant; 0.1 < *P* < 0.15 was considered a trend. **A**, Effect of adding fat on number of ADG of piglets (total); **B**, Effect of adding fat on ADG of piglets at hyper temperature; **C**, Effect of adding fat on ADG of piglets at thermoneutral temperature.

We analyzed 17 papers (47 treatments) to evaluate effects of supplemental fat on litter growth performance from birth to weaning using 1614 observations in control and 1620 observations in fat-supplemented treatments. Daily weight gain of litters was not different between control and fat treatments (*P* = 1.00; Fig. 6A). Likewise, levels of fat supplementation (< 10% or ≥ 10%) had no effect on litter ADG (data not shown).

We then segmented these treatments into two subgroups regardless the level of fat inclusion according to environmental temperature with 1265 observations in high temperature group (636 in control; 629 in fat treatment) and 1969 observations in neutral temperature group (978 in control; 991 in fat treatment). In high temperature, adding fat improved litter ADG from birth to weaning by 0.10 kg/d (95%-CI: 0.01 to 0.19 kg/d, *P* = 0.03; Fig. 6B). But in neutral temperatures, adding fat displayed no significant effect (*P* = 0.71; Fig. 6C).

### Survival rate to weaning

**Fig. 7:**
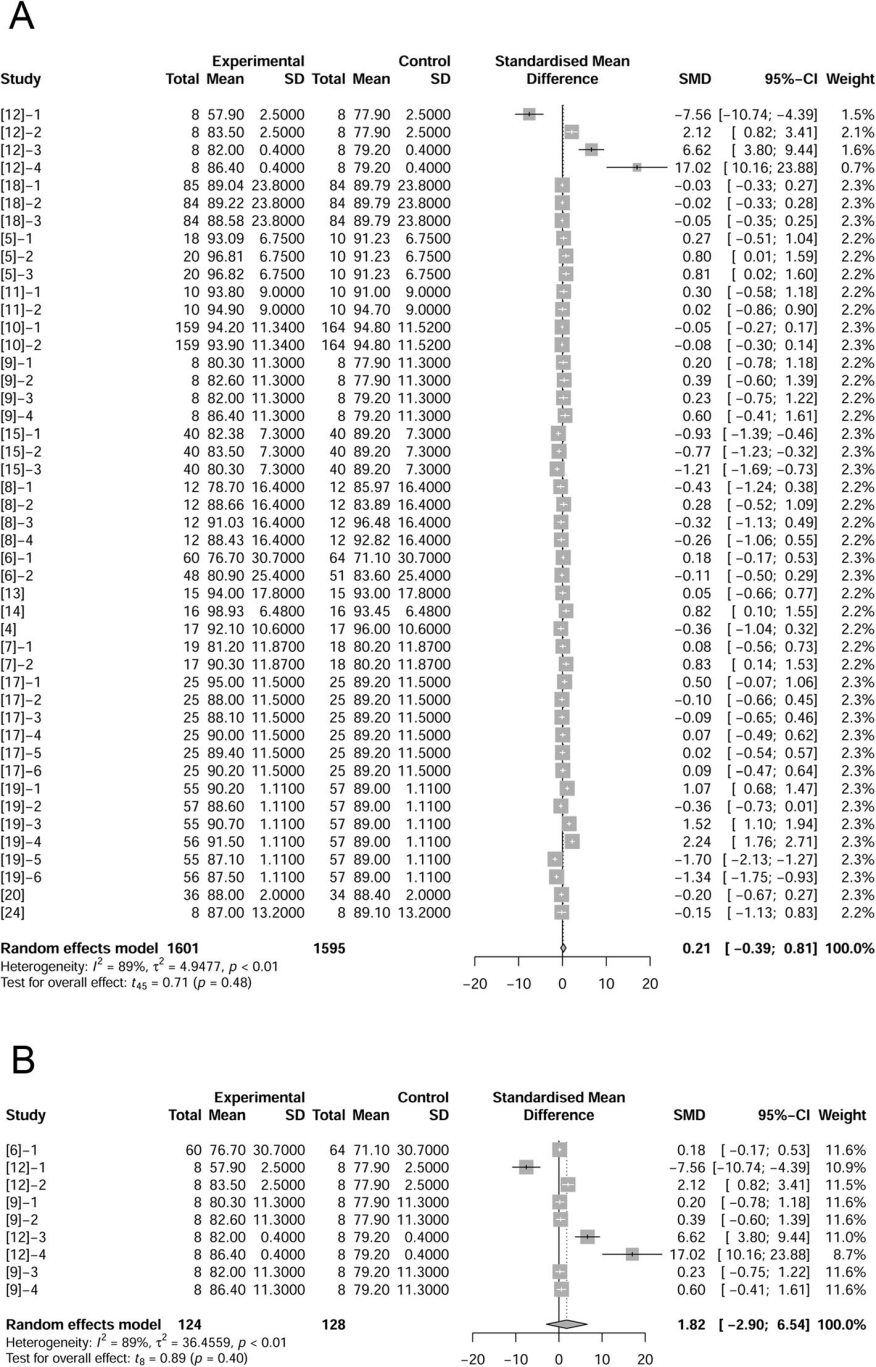
Forest plot showing effects of adding fat to diets during late gestation and lactation on survival rate (%) of piglets from birth to weaning. Figure lists treatments in studies with treatment No. (Study), treatment group of fat-treatment or un-supplemented control (Experimental vs. Control), total observations (Total), mean of the treatment and standard deviation (Mean, SD), standardized mean difference (SMD), 95% confidence interval (95%-CI) and the weight accounting for the total statistics (weight). ADG, average daily gain. Test results for heterogeneity and overall fat supplementation effect are listed at lower left of each figure. *P* ≤ 0.10 was considered significant; 0.1 < *P* < 0.15 was considered a trend. **A**, Effect of adding fat on number of survival rate of piglets (total); **B**, Effect of adding fat in sow diets on survival rate of piglets when survival rates were less than 80%.

We analyzed survival rate of piglets from birth to weaning. Authors of 17 papers (45 treatments) reported survival rate of piglets (1595 observations in control and 1601 observations in fat treatment). We observed no significant difference between control and fat treatments (*P* = 0.48, Fig. 7A). Previous researchers reported that fat can improve survival rate in herd with low piglet survival [33]. We thus examined effects in studies that reported piglet survival less than 80% in the controls (Fig. 7B). No significant differences were observed between control and fat supplementation treatments (*P* = 0.40). Likewise, piglet survival was not affected when considering the level of fat supplementation (2%, 4% and 10%; data not shown). In our dataset, fat supplementation in sow diets had no effect on piglet survival rate from birth to weaning.

### Milk fat concentration

**Fig. 8:**
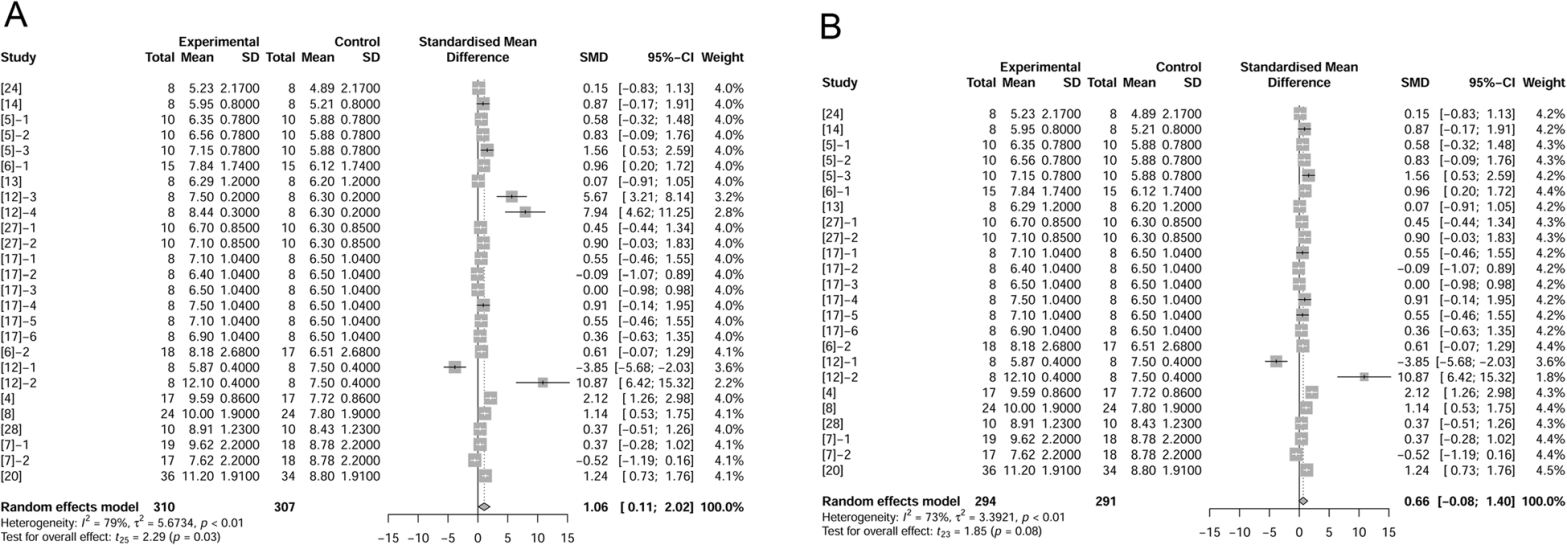
Forest plot showing effects of adding fat to diets during late gestation and lactation on milk fat concentration (%). Figure lists treatments in studies with treatment No. (Study), treatment group of fat-treatment or un-supplemented control (Experimental vs. Control), total observations (Total), mean of the treatment and standard deviation (Mean, SD), standardized mean difference (SMD), 95% confidence interval (95%-CI) and the weight accounting for the total statistics (weight). Test results for heterogeneity and overall fat supplementation effect are listed at lower left of each figure. *P* ≤ 0.10 was considered significant; 0.1 < *P* < 0.15 was considered a trend. **A**, Effect of adding fat in sow diets on milk fat content (total); **B**, Effect of adding fat on milk fat content at thermoneutral temperature.

Authors of 13 papers (25 treatments) reported fat concentration in milk. Milk was sampled from d 3 to 21 postpartum. There were 307 observations in control and 310 observations in fat treatment. Dietary fat supplementation increased the milk fat concentration by 1.06% (*P* = 0.03, Fig. 8A) compared to the control at all temperatures. When we removed 2 treatments which were conducted at high temperature and showed positive effects, the remaining 23 treatments (291 observations in control and 294 observations in fat treatment) still demonstrated increased milk fat concentration by 0.66% as a result of adding fat (*P* = 0.08, Fig. 8B).

### Losses of body weight and backfat thickness

**Fig. 9:**
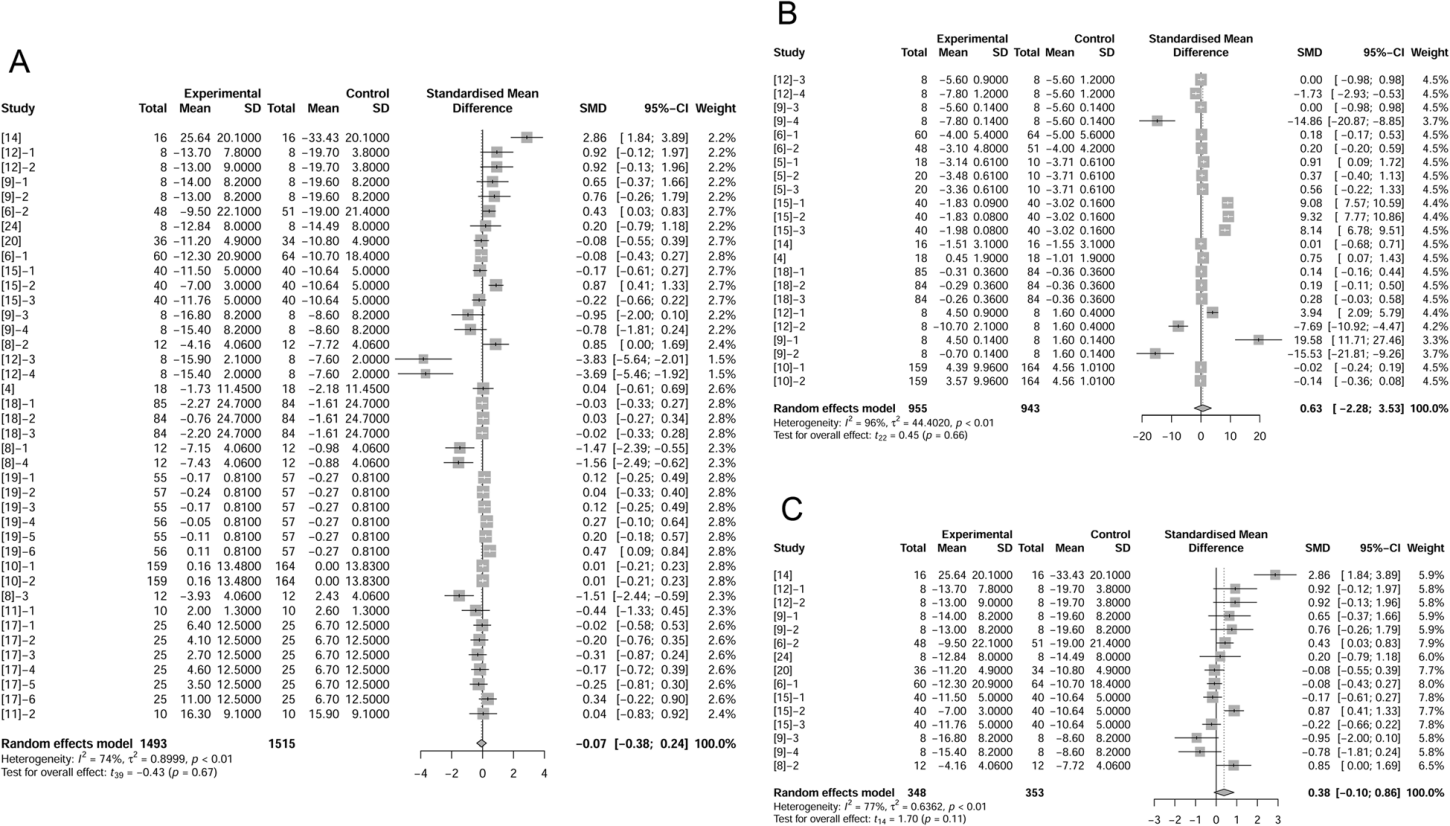
Forest plot of showing the effect of adding fat to diets during late gestation and lactation on losses of sow body weight (kg) and backfat thickness (mm). Figure lists treatments in studies with treatment No. (Study), treatment group of fat-treatment or un-supplemented control (Experimental vs. Control), total observations (Total), mean of the treatment and standard deviation (Mean, SD), standardized mean difference (SMD), 95% confidence interval (95%-CI) and the weight accounting for the total statistics (weight). Test results for heterogeneity and overall fat supplementation effect are listed at lower left of each figure. *P* ≤ 0.10 was considered significant; 0.1 < *P* < 0.15 was considered a trend. **A**, Effect of adding fat on the alteration of sow body weight; **B**, Effect of adding fat on sow backfat thickness; **C**, Effect of adding fat in sow diets on the greatest body weight loss of sows.

Dietary fat in sow diets resulted in reducing body weight loss and backfat loss during lactation [8, 9, 12, 15]. But according to our analysis based on 39 treatments (1515 observations in control and 1493 observations in fat treatment) for body weight change and 22 treatments (943 observations in control and 955 observations in fat treatment) for backfat thickness change, no significant effects of supplemented dietary fat were present (*P* = 0.67 and *P* = 0.66 respectively, Fig. 9A, B) by adding fat in diets. We extracted treatments where control sows lost more than 7.72 kg throughout lactation. These sows represented about 36% of the dataset with the greatest weight loss. In these higher weight-loss sows, fat supplementation tended to reduce lactational body weight loss by 0.38 kg (*P* = 0.11, Fig. 9C). However, loss of backfat thickness was not influenced by fat supplementation in these sows (data not shown).

### Wean to estrus interval (WEI)

**Fig. 10:**
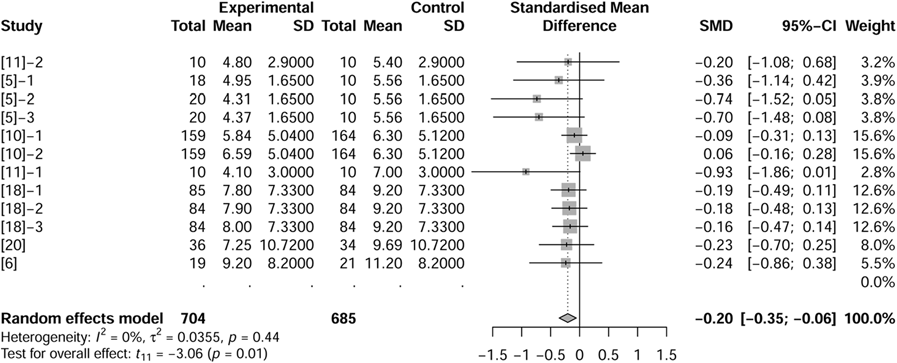
Forest plot showing effects of adding fat to diets during late gestation and lactation on WEI (d) of sows. Figure lists treatments in studies with treatment No. (Study), treatment group of fat-treatment or un-supplemented control (Experimental vs. Control), total observations (Total), mean of the treatment and standard deviation (Mean, SD), standardized mean difference (SMD), 95% confidence interval (95%-CI) and the weight accounting for the total statistics (weight). WEI, wean to estrus interval. Test results for heterogeneity and overall fat supplementation effect are listed at lower left of each figure. *P* ≤ 0.10 was considered significant; 0.1 < *P* < 0.15 was considered a trend.

Wean to estrus interval was reported in 6 papers (11 treatments) and included 685 observations in control and 704 observations in fat treatment. Fat supplementation shortened WEI by 0.20 d (*P* = 0.01, Fig. 10).

Collectively, the literature discussed in this review indicated that fat supplementation can be beneficial for sow reproductive performance and litter growth performance (Table 3). Fat supplementation in sow diets during late gestation through lactation period decreased ADFI (*P* < 0.05) and tended to increase ADEI (*P* = 0.11). Litter weights at birth (*P* = 0.40) or weaning (*P* = 0.46) were not influenced by fat supplementation. Added fat increased total numbers of piglets at birth (*P* = 0.07), but had no effect on liveborn per litter (*P* = 0.90) or survival rate (*P* = 0.48) of piglets to weaning. Fat supplementation had no effect on reducing loss of sow body weight (*P* = 0.67) or backfat thickness (*P* = 0.66), but did increase milk fat concentration (*P* = 0.03) and shorten WEI (*P* = 0.01). In some circumstances, fat supplementation had specific effects: it had a trend on improving the growth performance of piglets with light litter weights at birth (*P* = 0.14), or when the sows lost large body weight during lactation (*P* = 0.11). When the level of supplemented fat was 10% or greater, liveborn per litter decreased (*P* = 0.10) at neutral temperature.

**Table 3:** Overall effects of adding fat to sow diets on performance of sows and piglets^1^

Criterion	Source^2^	SMD^3^	95%-CI^4^	*P*-value^5^
ADFI, kg/d	Fig.1A	-0.14	-0.27; -0.01	0.04
ADEI, Mcal/d	Fig.2A	0.11	-0.03; 0.26	0.11
Litter weight (birth), kg	Fig.3A	0.09	-0.13; 0.30	0.40
Litter weight (weaning), kg	Fig.3B	-0.14	-0.51; 0.24	0.46
Litter size (birth)	Fig.4A	0.45	-0.03; 0.92	0.07
Liveborn litter size	Fig.5A	0.01	-0.11; 0.13	0.90
Litter-ADG, kg/d	Fig.6A	0.12	-0.21; 0.45	0.47
Weaning survival rate, %	Fig.7A	0.21	-0.39; 0.81	0.48
Milk Fat, %	Fig.8A	1.06	0.11; 2.02	0.03
Sow BW Loss, kg	Fig.9A	-0.07	-0.38; 0.24	0.67
Sow BF Loss, mm	Fig.9B	0.63	-2.28; 3.53	0.66
Sow WEI, d	Fig.10	-0.20	-0.35; -0.06	0.01

Note: ^1^ Overall effects of adding fat to sow diets on performance of sows and piglets. ^2^ Position of the figures where the relevant indicator data in the same row. ^3^ *SMD*: Standardized mean difference. ^4^ 95%-CI: 95% confidence interval. ^5^*P*-value: Probability values less than or equal to 0.10 were deemed significant and *P* values over 0.10 but less than 0.15 were considered a trend. Abbreviations: ADFI: Average daily feed intake during lactation period; ADEI: Average daily energy intake during lactation period; Litter weight (birth): Litter weight of piglets at birth; Litter weight (weaning): Litter weight of piglets at weaning; Litter size (birth): Total piglet numbers per litter at birth; Liveborn litter size: Total live piglet numbers per litter at birth; Litter-*ADG*: Litter average daily gain during lactation; Sow *BW* Loss: Loss of body weight loss of sows from farrowing to wean; Sow *BF* Loss: Loss of backfat thickness of sows from farrowing to wean; Sow *WEI*: Weaning to estrus interval of sows

## Discussion

Sows lose significant amounts of energy during lactation. Increased body weight loss has become a greater problem in sows with larger litters [34, 35]. A recent comparison demonstrated that porcine fetuses are 40% heavier and milk yield increased by 4 folds between 1935 and 2010 [36]. Sufficient dietary energy intake of sows can endorse the energy supply to piglets via milk and energy storage for subsequent estrus cycles. Thus, the primary purpose of adding fat to sows’ diets is to increase energy intake of sows by increasing energy density of the diet. In this review, fat supplementation consistently reduced ADFI and tended to increase ADEI between control and fat-fed sows. Regulation of appetite and the resulting feed intake is an integrated scenario of several factors including the ingested nutrients and hormones [37, 38].

Ingestion of nutrients triggers release of a series of hormones from gastrointestinal tract. In pigs, these hormones are mainly cholecystokinin (CCK), glucagon like peptide-1 (GLP-1), peptide tyrosine tyrosine (PYY) and ghrelin [39]. CCK, GL and PYY elicit satiation of food. A high-fat meal can effectively induce secretion of these satiety hormones compared to high-starch diets [40]. In addition, previous studies reported that pigs fed with high fat diets resulted in changes in regulatory neuropeptides in the hypothalamus and alterations mostly in the dopaminergic system in the ventral hippocampus [41]. Ghrelin is unique among gastrointestinal hormones because it is a hunger signal. Ghrelin can be suppressed by ingested food especially food with high caloric density [42]. Carbon chain length and saturation of fatty acids impacts the effect of dietary fat on appetite and releasing of satiety hormones [43–47]. Fatty acids with longer carbon chain lengths had stronger effects on stimulation of appetite compared to shorter chain lengths of carbons (e.g., C16 > C10). The effect of polyunsaturated fatty acids was higher than that of monosaturated fatty acids. Hormones involved in regulation of feed intake integrate with plasma glucose, insulin, intestinal osmolality and enteric neurons to maintain a balance of energy intake and energy homeostasis in the body [48]. In lactating sows, adipocyte produced leptin contributed to the regulation of feed intake. Serum leptin levels were positively correlated with backfat thickness [49]. Previous studies have reported that serum leptin levels decreased by day 7 of lactation [50] and reached its lowest point during peak lactation [51]. Therefore, serum leptin may only play an important role in early lactation. Circulating leptin, luteinizing hormone concentrations and feed consumption during lactation are influenced by dietary energy intake during lactation in sows [52].

Under tropical temperatures, fat supplementation of diets increased feed intake and consequently increased energy intake according to our analysis. The additional fat intake increased the liveborn per litter and piglet growth during lactation. There were not enough observations to analyze how the additional fat intake affects changes of body weight, backfat thickness and WEI in sows at tropical environment. Higher feed intake of sows under heat stress may be due to a lower heat increment of fat compared to other nutrients [53]. Our analysis verified that fat supplementation was beneficial for sow feed intake, energy intake, and piglet growth performance under the condition of heat stress.

Fat contains 2.25 times more energy per unit of weight than carbohydrates. Vegetable oils are higher in ME than animal fats [54]. Dietary fat elicits several positive effects including improved palatability [55], reduced feed consumption, and improved feed efficiency [56]. Dietary starch supplementation in lactating primiparous sows functions in protein deposition in piglets while dietary fat is used preferentially for milk fat synthesis at a high feeding level [57].

Moreover, more than as an energy source, fat plays an important role in promoting reproductive functions. Other researchers demonstrated that sows fed with fat supplemented diet had higher piglet survival rate, increased growth rate and shorter postweaning interval to estrus than those sows fed with iso-energetic diets that relied on starch to provide metabolizable energy [58, 59]. In the present summary, fat supplementation clearly shortened WEI. Fat has long been deemed as a nutritional and metabolic regulator of reproduction in sows (reviewed in [60]). In women, mice, and rats, diets enriched with fat increased ovarian steroids (estradiol and progesterone) in circulation [61–63]. Thus, fat is likely involved in the induction of post weaning ovulation and shortening of WEI.

Ovulation is closely related to body energy intake and is controlled by the hypothalamus-pituitary-ovarian axis. There are two theories explaining the correlation between energy balance and reproduction. One is the metabolic fuel hypothesis which proposes nutrient molecules and metabolites can be oxidized and act as sensory stimuli for the responses of reproductive axis [64]. The other theory proposes that fat’s promoted effect on the production of estrogen production and sex hormone binding globulin. Fat supplementation improves the production of estrogen and sex hormone binding globulin, and these products can elevate the sensibility of hypothalamic-pituitary-ovarian axis [65]. The preservation of reproductive function relies on a certain amount of adiposity [66]. Therefore, fat can act as both metabolic fuel and adipose preservation to regulate reproductive functions.

According to the analysis in this article, fat supplementation did not change losses of body weight and backfat thickness during lactation. Milk fat concentration was increased by adding fat to the sow’s diet but piglet ADG was not improved.

One of the reasons that increased concentration of milk fat didn’t increase ADG of piglets was likely due to the reliance on cross-fostering rather than insufficient digestion of milk fat by piglets. Around 98% of commercial pig farms use cross-fostering as a management technique for creating litters with more uniform body weight [67]. Cross-fostering should be performed as early as possible, usually from 12 to 24 h after farrowing when the teat order is not established [68]. Cross culture is usually adjusted within a treatment. Researchers reported that neonatal piglets had a much higher capacity to absorb fat than milk provided [69], which greatly exceeded the output capacity of sow mammary gland. Different cross-fostering strategies could affect the growth performance of litters. For example, litter growth performance was decreased if piglets with lighter birth weights were cross fostered with heavier piglets, or new born piglets with heavier body weights suckled middle and posterior teats [70], or cross foster conducted later than 48 h. after farrowing that caused higher plasma cortisol [71]. Litter composition, cross foster time point, body weight variation of litter and access to creep feed are all variables for determination of piglet ADG.

We expected piglet survival rate from birth to weaning to be improved especially since adding fat increased colostral fat content [14, 72], which was critical for new born survival [73]. Likewise, we found no significant effect of fat on piglet survival rate in studies that reported survival rates lower than 80%. Pettigrew and Moser reviewed studies during 1974 to 1979 and found that adding fat to sow diets improved piglet survival rate in herds with lower than 80% [33]. If the piglet survival rate was above 80%, fat supplementation had little effect on improving survival rate. In the current article, collected studies from 1986 through 2020. During two to three decades since Pettigrew and Moser’s report in 1991, sows have undergone a series of genetic selection, and pig farm facilities and management have greatly improved. Overall survival rates in studies summarized for our analysis ranged from 71.10% to 96.48%. Only 17% of studies reported survival rates below 80% in our collection. With survival rate at such a high level, sows were not responsive to dietary fat such that survival rate was not affected.

In modern era, sow prolificacy results in larger litters but it also increases the proportion of piglets born with low body weights [70]. Adding fat in sow diets further increased the litter size but the total litter weight at birth was not changed, neither was liveborn per litter, which suggested increased incidence of stillborn, mummied, and dead piglets at birth.

The conclusions drawn in this paper were derived from the overall analysis of 19 papers rather than an individual study. Under a specific circumstance, types of oil/fat (digestible energy of a specific fat, ratio of unsaturated to saturated fatty acids, carbon chain length [74]), environmental temperature, supplementation level of fat, parity of sows and management strategy can all impact on the effect of fat supplementation. Additional, new functions of dietary fat could be revealed by meta-analysis with more studies in the future.

## Conclusions

We reviewed 19 papers published from 1986 to 2020 and determined that compared to un-supplemented controls, adding fat in sow diets during late gestation and lactation decreased ADFI (*P* < 0.05) and tended to increase ADEI (*P* = 0.11). Fat supplementation had no effect on litter weights at birth (*P* = 0.40) or weaning (*P* = 0.46). Total numbers of piglets per litter at birth were increased by fat supplementation (*P* = 0.07), but we observed no effects on liveborn per litter (*P* = 0.90) or survival rate (*P* = 0.48) of piglets to weaning. Fat supplementation had no effect on sow body weight loss (*P* = 0.67) or backfat thickness changes (*P* = 0.66), but increased milk fat concentration (*P* = 0.03) and shorten WEI (*P* = 0.01). In specific circumstances, fat supplementation tended to improve growth performance of piglets with low litter weights at birth (*P* = 0.14), or when sows lost large amounts of body weight during lactation (*P* = 0.11). When the level of supplemented fat was 10% or higher, it decreased the liveborn per litter (*P* = 0.10) at neutral temperature. It can be concluded that during late gestation and lactation, the strategic use of fat could be beneficial for sow reproductive performance and litter growth performance.

## Supplementary Information

**Additional file 1.** Relationship between fat level and SMD. A, Fat level and SMD of ADFI (Fat treatment vs. un-supplemented control), kg/d; B, Fat level and SMD of ADEI (fat treatment vs. un-supplemented control), Mcal/d. SMD: Standardised mean of difference. Diameters of bubbles represented weighing of SMD.

**Additional file 2.** Regression analysis on the level of fat supplementation and increased ME (Mcal). X axis: Level of added fat; Y axis: Difference of ME of diets between added fat and un-supplemented control (Mcal). R linear repression (Pearson’s) was performed. The regression equation was: 4.89 * Fat level-0.01614=Difference of ME R-squared=0.7792.

## Data Availability

Not applicable.

## Abbreviations

ADEI: Average daily energy intake
ADFI: Average daily feed intake
ADG: Average daily gain
CCK: Cholecystokinin
CI: Confidence interval
DE: Digestible energy
GLP-1: Glucagon like peptide-1
ME: Metabolizable energy
SMD: Standardized mean difference
PYY: Peptide tyrosine tyrosine
WEI: Wean to estrus interval
