# Supplementary figures and images for: A systematic review and meta-analysis of dietary fat effects on reproductive performance of sows and growth performance of piglets
Source: J Anim Sci Biotechnol. 2022 Feb 8;13:12. doi: 10.1186/s40104-021-00662-3 (PMC8822652; doi:10.1186/s40104-021-00662-3)

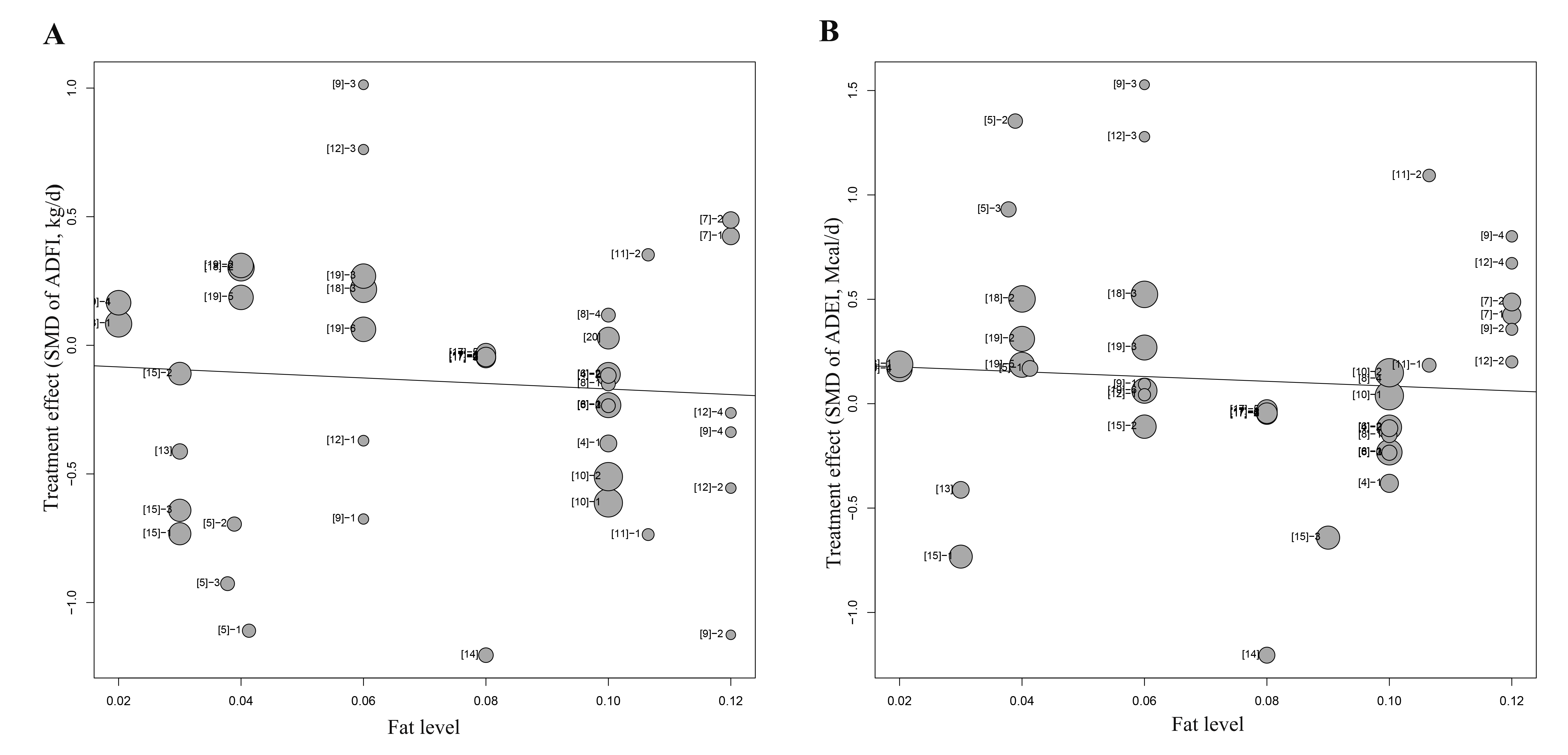

Supplement: Supplementary file 1 — Additional file 1. Relationship between fat level and SMD. A, Fat level and SMD of ADFI (Fat treatment vs. un-supplemented control), kg/d; B, Fat level and SMD of ADEI (fat treatment vs. un-supplemented control), Mcal/d. SMD: Standardised mean of difference. Diameters of bubbles represented weighing of SMD. [file 40104_2021_662_MOESM1_ESM.tif]

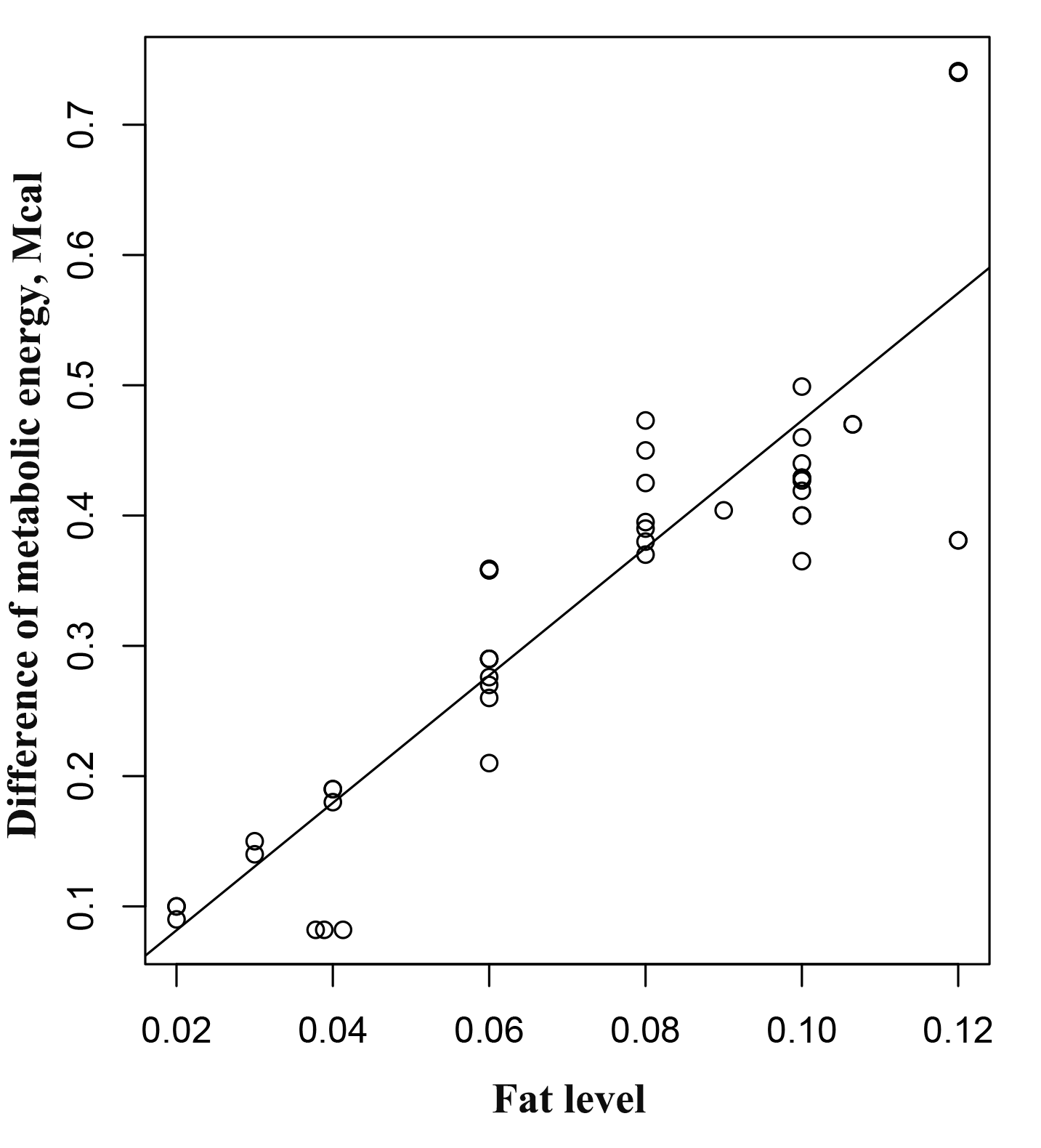

Supplement: Supplementary file 2 — Additional file 2. Regression analysis on the level of fat supplementation and increased ME (Mcal). X axis: Level of added fat; Y axis: Difference of ME of diets between added fat and un-supplemented control (Mcal). R linear repression (Pearson’s) was performed. The regression equation was: 4.89 * Fat level-0.01614=Difference of ME R-squared=0.7792. [file 40104_2021_662_MOESM2_ESM.tif]
